# IT and the Quality and Efficiency of Mental Health Care in a Time of COVID-19: Case Study of Mental Health Providers in England

**DOI:** 10.2196/37533

**Published:** 2022-12-29

**Authors:** Frederick Hassan Konteh, Russell Mannion, Rowena Jacobs

**Affiliations:** 1 Health Services Management Centre School of Social Policy University of Birmingham Birmingham United Kingdom; 2 Centre for Health Economics University of York York United Kingdom

**Keywords:** COVID-19, mental health care, information technology, digital, inequalities, sociotechnical systems

## Abstract

**Background:**

In England, COVID-19 has significantly affected mental health care and tested the resilience of health care providers. In many areas, the increased use of IT has enabled traditional modes of service delivery to be supported or even replaced by remote forms of provision.

**Objective:**

This study aimed to assess the use and impact of IT, in remote service provision, on the quality and efficiency of mental health care during the pandemic. We drew on sociotechnical systems theory as a conceptual framework to help structure the gathering, analysis, and interpretation of data.

**Methods:**

We conducted a national scoping survey that involved documentary analysis and semistructured interviews with 6 national stakeholders and case studies of 4 purposefully selected mental health providers in England involving interviews with 53 staff members.

**Results:**

Following the outbreak of COVID-19, mental health providers rapidly adjusted their traditional forms of service delivery, switching to digital and telephone consultations for most services. The informants provided nuanced perspectives on the impact on the quality and efficiency of remote service delivery during the pandemic. Notably, it has allowed providers to attend to as many patients as possible in the face of COVID-19 restrictions, to the convenience of both patients and staff. Among its negative effects are concerns about the unsuitability of remote consultation for some people with mental health conditions and the potential to widen the digital divide and exacerbate existing inequalities. Sociotechnical systems theory was found to be a suitable framework for understanding the range of systemic and sociotechnical factors that influence the use of technology in mental health care delivery in times of crisis and normalcy.

**Conclusions:**

Although the use of IT has boosted mental health care delivery during the pandemic, it has had mixed effects on quality and efficiency. In general, patients have benefited from the convenience of remote consultation when face-to-face contact was impossible. In contrast, patient choice was often compromised, and patient experience and outcomes might have been affected for some people with mental health conditions for which remote consultation is less suitable. However, the full impact of IT on the quality and efficiency of mental health care provision along with the systemic and sociotechnical determinants requires more sustained and longitudinal research.

## Introduction

COVID-19 has negatively impacted mental health worldwide, and the full ramifications of the pandemic on population mental health may last much longer than the initial phases of the pandemic [1,2]. Deterioration in existing mental health and new presentations are likely to contribute to the rising demand for mental health services, including depressive and anxiety disorders, which may stem from the psychological and economic effects of the pandemic linked to factors such as bereavement, restrictions in movement, lack of social interactions, domestic violence, and unemployment [2,3]. In England, as in most parts of the world, the pandemic, especially in the initial phase, severely disrupted health and social care provision, including mental health services [1,4,5]. By April 2021, the demand for mental health services in England was at a record high without a commensurate increase in funding for the sector [6]. With the initial focus being primarily on physical health (fighting the pandemic), COVID-19 has challenged the resilience of mental health providers, requiring them to adapt and transform their service delivery.

COVID-19 hit shortly after the National Health System (NHS) Long Term Plan for England was published, at the heart of which was a strong commitment by the UK government to use digital technology to transform health and social care [7,8]. In the United Kingdom, especially England, the use of technology in health care was relatively low before the pandemic—remote consultations were predominantly via telephone [9-12] with community mental health services experiencing the least investment in new technology [13]. At the national policy level, there was an acknowledgment that poor technological infrastructure in health and social care needs to be addressed [14].

The NHS Long Term Plan articulates the government’s strategic intention to digitalize health and care in England, offering patients the choice to access web-based services as an alternative to face-to-face consultations [7]. System interoperability is a key aspiration of the Long Term Plan, with the potential for significant cost savings for the NHS, saving time and money for service users, and the convenience of telephone and video consultation. The NHS Mental Health Implementation Plan, an offshoot of the NHS Long Term Plan, outlines the provision of digital options to service users and the rolling out of “digitally-enabled models of therapy” for “specific mental health pathways” by 2021-2022 and for local systems to make use of “digital clinical decision-making tools” by 2023-2024 [13]. COVID-19 has helped to fast-track the NHS Long Term Plan’s digital technology actualization. In a policy statement at the start of the pandemic (July 30, 2020), the Department of Health and Social Care in England urged providers to effect a wholescale switch to remote service provision through the use of telephone and digital technology [10]. Mental health providers appeared to have responded to this call. Thus, the pandemic has accelerated the rapid rollout of IT in mental health service delivery [1,4,11,15,16].

Research has highlighted the unprecedented use of remote (digital and telephone) consultations in mental health care during the pandemic [1,4,5,9-12,15-20]. The greatest benefit was that it enabled providers to respond flexibly to the needs of service users while adjusting to pandemic-enforced challenges and restrictions. In the absence of face-to-face consultations, the use of IT facilitated a higher frequency of contact between professionals and patients than would have hitherto been possible [1,4,5,11,16]. Evidence that telephone has remained the most common means of remote consultation for mental health care providers in England, throughout the pandemic [10,11], suggests that the country has some catching up to do with respect to digital technology in mental health.

One of the benefits of remote consultation is its potential to overcome geographic barriers (the friction of distance), allowing access to populations in remote locations with serious accessibility problems, thereby reducing inequalities. However, a serious disadvantage is the potential for digital exclusion among vulnerable and disadvantaged population groups [1,10,11,18,21]. For example, technology and environment-related factors, such as problems with broadband or internet connectivity, continue to render many people living in remote locations relatively disadvantaged. Linked to digital exclusion is digital poverty, which encompasses issues of affordability (of technological devices and facilities including internet connection), accessibility, skills, and motivation [18].

The COVID-19 pandemic has witnessed a growing research interest in the use of IT in health care delivery. However, as Ellis and others [5] observed, empirical research has focused largely on physical health, whereas the mental health sector remains underresearched. There has been a call for research to examine the factors that influence the optimal use of technology in health care delivery, generally [9]. The purpose of this study was to understand how mental health providers in England used IT, including internet-enabled digital applications and traditional forms of remote service delivery, during the pandemic and the implications of this shift for quality and efficiency. It also seeks to enhance the understanding of the complexity of factors (social or human, system-related, and technical) affecting the use of IT in remote mental health care delivery and to apply the sociotechnical systems (STS) framework [22-25] to a case study design.

## Methods

### Theoretical Framework

**Figure 1:**
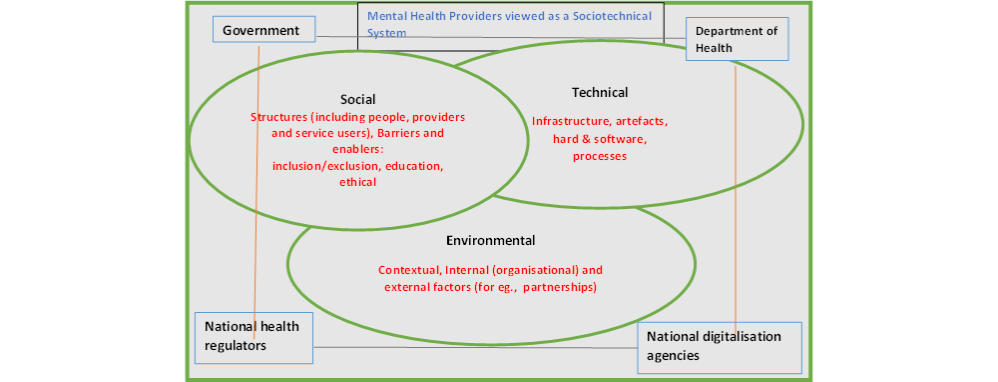
Sociotechnical systems and IT in mental health care provision.

An important notion related to the use of IT in health care is that, given their multiple objectives and complexity, health care organizations are best conceived as STS [22-25]. We applied the STS theory during data analysis, helping to interpret the research findings and to enhance the understanding of the major factors at work in the use of technology in mental health service delivery [22,25]. On the basis of the STS model, a health care system, like any complex and dynamic system, comprises a mix of interacting social and technical subsystems that affect each other [22-26]. Figure 1 illustrates the adaptation of the STS framework used in this study. Scholars have proposed a variety of STS models [27]. One model suggests that STS is composed of 2 sets of variables or subsystems, namely, social and technical structures [26]. The technical subsystem comprises the process and technology components, whereas the social structures represent people (including program specialists, service providers, and service users) and organizations (including groups, teams, and departments). Another conceptualization of STS is that it comprises social, technical, and environmental components [28]. STS aligns with the key tenets of general systems theory and the notion of a nonlinear complex system [28]. STS theories emphasize the interdependence of the subsystems and the need for joint optimization, which requires an alignment of social and technical constituents with a wider environment or system to achieve the desired transformational goal of an organization [22-28]. The proposed framework (Figure 1), an adaptation of the STS, includes social, technical, environmental, and contextual factors as well as the wider systemic influences, including government, the Department of Health, and regulatory or facilitating agencies (notably NHS Digital).

### Research Questions

This study is derived from a larger study that aimed to understand how mental health trusts in England adapted and adjusted services to cope with the COVID-19 pandemic [29]. Our interrelated research questions were as follows:

- How have mental health providers adapted service provision and used technology in remote service delivery during the pandemic?
- What were each provider’s technological capabilities, such as allowing adjustments to service provision?
- How has the use of technology affected the quality and efficiency of mental health care delivery during the pandemic?

### Study Design

We used a qualitative research approach in a multiple case study design, comprising 4 mental health providers, renamed here, to protect anonymity, as providers A, B, C, and D. The case study has long been considered suitable for the in-depth investigation of a contemporaneous subject [30]. The multiple case studies and qualitative research approach allowed for comparisons of similarities and differences to be explored between cases, with the scope to uncover new variables in a complex social context, where the researchers could not “manipulate” the subjects of inquiry [30-32]. At the outset, we conducted a scoping survey, comprising a review of relevant documentation and key informant interviews with key staff from national stakeholder organizations.

### Data Collection

Data were collected between March and December 2021. In the scoping phase, we emailed 10 national stakeholder organizations, explaining the study and requesting contact with a suitable informant. These were purposively selected based on their role in the national health system or mental health care oversight. With 60% success, this resulted in interviews with people who held very senior or executive-level positions in the following organizations: NHS England (2), the Care Quality Commission (CQC; 1), the Mental Health Commissioners Network (MHCN; 1), the Health Care Financial Management Association (1), and the *Get it Right First*-*Time* program (1). Not only do their perspectives help in grounding our research within extant national policy developments and interests around the use of digital technology in health care delivery in England, but they also provide complementary evidence, especially for the key generic findings of the case study.

The 4 case study sites were purposefully selected to represent the opposite ends of the performance spectrum (based on our earlier work in which the research team used relevant secondary NHS data to rank all mental health providers in England into low- and high-performance categories). The selected providers offer a range of services and reflect different population catchments, staff sizes, and geographic spread (2 [A and B] mainly rural, 1 [C] metropolitan, and 1 [D] rural or urban based). The research team conducted 53 semistructured interviews with 72 potential informants (18 at each site) who were invited to participate (with a response rate of 74%). Participants, who were purposively sampled, comprised executive team members, senior managers and service directors or clinicians, patient representatives, and clinical commissioning groups responsible for commissioning services for each provider. For a breakdown of study participants by organization refer to Multimedia Appendix 1. Emails were sent out by the designated research support officer at each site, inviting eligible staff members to the study and introducing the researcher (interviewer). A web-based meeting was arranged for those who expressed a willingness to participate in the study. A consent form, signed by the researcher, was emailed within 6 to 24 hours before the interview, and the informant was encouraged to read, sign, and return via email before the interview. Most of the interviews were conducted using Microsoft Teams, and in a few cases (mainly with patient representatives), interviews were conducted using Zoom (Zoom Video Communications). All informants were asked for permission to record the interview (even though they would have indicated their approval on the consent form). Interviews lasted between 30 and 45 minutes; the questions for national stakeholders and case study informants cover similar themes but with slight modification for the latter; the themes or questions span beyond the focus of this paper—being part of a larger study (refer to Multimedia Appendix 2 for the topic guides).

### Data Analysis

As reported in our previous publications [33], this was part of a larger study in which we used administrative and patient survey data for each mental health provider in England to construct a composite performance indicator (ranking providers into low- and high-performing categories). Documents from the providers’ websites and relevant national reports were also reviewed, providing useful background and policy-related material to complement the data from the case study interviews.

We used NVivo (QSR International) to code the transcripts and analyze the data. We coded and analyzed all transcripts from the interviews as one file, both within and across cases, enabling us to explore differences and synergies between and across providers and national stakeholders [31,32]. We followed the 5 stages of the framework method (familiarization, theme identification, indexing, charting, and interpretation) to analyze and structure the data [34]. On the basis of abductive theorizing and pattern matching, we explored the views and experiences of participants regarding our study objectives, allowing us to draw out and integrate the common themes from the interview data. The codebook was developed by FK and RM and reviewed by RJ, and coding was carried out by FK, with coding outputs shared with other authors for discussion. For the NVivo codebook, please refer to Multimedia Appendix 3.

### Ethical Considerations

We obtained research ethics approval from the NHS Health Research Authority (reference number: 21/PR/0047) and the participating organizations. There was an undertaking (stated in the research passport) that the research does not involve “regulated activity with children and/or adults as defined in the Safeguarding Vulnerable Groups Act 2006.” In addition, in the Integrated Research Application System form, it was stated that the study has nothing to do with “human tissue samples and data.”

Informed and written consent was obtained before every interview signed by the researcher and countersigned by study participant. The participant’s information sheet assured every participant about confidentiality and anonymity of the information they would provide and their identity. It stated further that participation was voluntary, and as the research was not considered to be harmful, there were no compensation arrangements. Participants were also advised about where to report if they had any concerns or complaints regarding the study.

## Results

### Providers Adapting Service Provision by Switching to Remote Service Delivery Following the Outbreak of COVID-19

There was a convergence of views from informants that following the outbreak of COVID-19, mental health providers switched to remote service provision via telephone and video consultations at an unprecedented speed and scale. Providers adopted web-based platforms and digital apps to ensure contact with patients and web-based interaction among staff, particularly during periods when COVID-19–imposed restrictions made physical interaction impossible. Safety considerations for both health professionals and patients were part of the motivation for the rapid switch to remote service provision. Study respondents were of the view that service users have benefited from services being “more responsive and immediate”:

It seemed to us that people adopted very quickly, much more quickly than usual. Normally if you’re going to roll out a new IT system they’re talking about it for four years...to get an electronic note system up and running and everybody using it. But this actually appeared to work really quickly...

CQC Official

...We took action pretty quickly to, as I’m sure others did, a) protect our staff by ensuring that they were working from home if they could but b) open up digital online and telephone channels...

Provider A, Director of Strategy

Digital consultation was not considered a viable option for some services until COVID-19 hit. For example, the Manager for Older People’s Community Mental Health Team in provider C noted that they have had “to offer e-consultations as an option which we weren’t really doing prior to COVID.” However, COVID-19 saw an upsurge in the use of digital apps such as Attend Anywhere, eConsult, and Kooth and web-based meeting platforms, particularly Zoom and MS Teams. It was suggested that providers adopt remote service provision as a first or default option when face-to-face contact was not possible. Informants made repeated references to “digital first” and “digital by default,” which resonated with the emphasis on the development of technological solutions set out in the NHS Long Term Plan. Despite what appeared to be a remarkable effort of mental health providers to escalate remote service provision, including digital options, during the pandemic, there was a perception among informants that telephone, which was generally viewed as inferior to video consultation, remained the most common medium of interaction between mental health providers and service users:

The adaption we’ve done, we do more video consultations now, Attend Anywhere...in the early stages we did group things on Zoom, on Teams...we’ve got Attend Anywhere now for one-to-one interventions

Provider B, Head of Mental Health & Learning Disability

The message we have given really has been digital first, not digital only. So, if it can move online, move it online...

Provider A, Director of Nursing

There is a bit of a myth about digital health, digital mental health. What they mean is they have been using the phone a lot. I mean 95% of this is by phone.

NHS England Official (2)

Some services, notably Increasing Access to Psychological Therapy, were able to deploy technology during the pandemic more rapidly than others because before the pandemic, they had already been ahead in the use of telephone and digital technology.

### Providers’ Technological Capabilities in Coping With COVID-19—Systemic Factors

The fact that telephone consultations have remained the most common form of remote interaction with service users means that there are still opportunities for digitalizing health care. However, technological capabilities varied across providers both before and during the pandemic. How technologically prepared a provider was depended greatly on its digital maturity, which in turn was a function of system-wide factors. Two of the providers were a global digital exemplar (GDE) and a GDE “fast follower” D and C, respectively (both high-performing providers). These were national awards that came with extra funding, which the National Health System set up in 2017 to support the development and use of digital technology among a selected number of NHS providers, following a bidding process. A GDE is similar to a pacesetter in the use of digital technology, and a GDE fast follower becomes affiliated with a GDE to collaborate and share best practices in the use of technology to deliver health services. This meant that providers D and C were investing optimally in technology and were relatively digitally mature and technologically prepared when COVID-19 arrived. In contrast, the rollout of digital technology was slower for the low-performing providers, A and B, who were less prepared technologically. Some informants in these 2 organizations reported a few challenges with the adoption of digital technology during the pandemic:

We’re really fortunate to be quite a digitally enabled organisation. So we already had numbers of virtual platforms...We were able to move probably more swiftly than others to a virtual platform.

Provider D, Director of Nursing

I think we weren’t terribly well prepared. I don’t think there was much...suddenly there was need for all clinicians to have a laptop, to have an “Attend Anywhere” account and be trained up in Attend Anywhere. We weren’t prepared or that and we were on the back foot.

Provider B, Medical Director

It emerged that all mental health providers, including those who were technologically less prepared, were boosted in their quest to repurpose services through additional funding from the government during the pandemic. This system-wide support has enabled providers to invest in different areas and may help minimize existing disparities with regard to resource allocation and the development of innovation and technology:

We have managed to get more funding into capital spend...in a lot of ways we have actually got more money going into mental health and we are more attuned to what is going on in the country, and that will carry on with the official investment over the next year.

NHS England Official (2)

A more flexible approach to contracting, in which the provider was allowed freedom to take quick decisions around spending, has also helped. Informants agreed that the collaboration and cooperation with partners, especially between providers and their Clinical Commissioning Group, was part of the reasons they managed to adapt service provision so quickly, including in digitalization:

I think the reason they got better was because we had a shared common purpose and we didn’t have the luxury of time to debate and discuss and argue and write papers and develop plans, and all that stuff. It was a matter of, we need to act and we need to act now and we need to act together...It was a matter of, we need to do something and do something different and do something quickly.

Provider B, CCG_P1 Deputy Director

Drawing further on system-wide collaboration, at least three of the case study providers benefited from private sector support. For example, provider B applied for and received a grant from Barclays Bank, which they used to supplement investments in digital technology. Providers A, B, and C worked with the voluntary sector in setting up internet cafes at strategic locations (to allow patients access to services remotely and free of cost), supporting others with digital gadgets, and with the basic skill of using digital technology.

### Technology Impact on the Quality and Efficiency of Mental Health Care Delivery During COVID-19—Sociotechnical Determinants

National stakeholders and case study informants provided mixed views about the impact of COVID-19 and the influence of technology on quality and efficiency. They pointed to the speed with which a health professional would make contact with several patients within a short period without having to travel. For example, the Director of Finance in provider B suggested that patients did not have to wait for long before being attended to, and this, along with the convenience of accessing service remotely, has had a positive effect on patient experience. This was said to have allowed mental health professionals time for other activities, for example, administration and work. In addition, it enabled health providers to reduce their carbon footprint and contribute meaningfully toward the government’s commitment to achieving a net zero NHS by 2045. Organization-wide meetings and internal team meetings quickly switched to video conferences, and these were reported to be very well attended.

With respect to quality, the perspectives of case study informants centered on the following: quality of mental health service has not been (adversely) affected; quality might have been affected, if not compromised, in certain instances; and it would take time for services to determine the actual impact on quality using the conventional indicators or measures. Informants were more assured in their assessment of how the use of IT during the pandemic has positively reflected on mental health care efficiency:

I think the quality and the ability to be able to see people, and digitally see people, has been an improvement, because we’ve probably been able to contact people maybe more frequently than we would have done if we were doing face-to-face.

Provider C, Director of Nursing

I think there’s probably a mixed picture. So, for those people who engaged well with digital their service was probably enhanced because it was a lot more responsive and immediate, and for those that didn’t or couldn’t then their provision would have been reduced especially in the early days. We kept the wards open the whole time for high acute and emergencies, but for community cases if you weren’t able to engage digitally then you probably did experience some deterioration or you certainly weren’t getting the same level of support that you were used to.

Provider A, Associate Director of People

What I would caveat on that is we don’t know the true quality benefits of digital working yet because we’ve got no objective measures at the moment to really understand that.

Provider A, Director of Operations

There was a notion that web-based interfacing, including conducting ward rounds via video technology, prevented people from congregating in confined spaces and therefore helped to minimize the spread of COVID-19. In addition, providers organized web-based visitations (meetings) between patients in inpatient wards and their families, which served as a good substitute for physical visits and helped to enhance patient experience during the pandemic. Some informants suggested that the inherent social and technical barriers, as presented below, would have compromised the quality or minimized the effectiveness of digital consultation. However, while drawing on funds provided by NHS England or grants from donor organizations or working with the voluntary sector, mental health providers did their best to address the problem of digital inequalities.

A range of social and technical factors can be seen to have a bearing on IT use during the pandemic, which is relevant to the STS framework. First, informants made repeated references to “digital poverty,” “digital divide,” “digital exclusion,” and “digital inequality,” suggesting that the scaling up of IT in service provision during the pandemic might have exacerbated inequalities. It has been suggested that the low socioeconomic status of some service users (or sheer poverty) affects their ability to afford or own a digital device or connect to the internet or Wi-Fi. Informants, especially of providers A and B, also noted that access to digital consultation might be a huge challenge for service users who reside in very remote locations. Digital illiteracy emerged as another factor driving digital exclusion; there was a suggestion that this was mainly a function of age and that older service users were less “tech-savvy” than their younger counterparts:

...Access has been quite problematic during the pandemic and certainly during lockdown because largely this has been virtual access...and not everybody finds that easy and there is a group of people who are digitally disadvantaged who find that problematic.

MHCN Official

Now, for the digitally excluded they [digital options] are useless...I am a consultant psychiatrist for a homeless team in [City X]. I have got two patients over the last year and I have been using the phone to speak to them. Most of the rest haven’t got a phone.

NHS England Official (2)

When people have said “I don’t like it, I can’t use it” and actually we still do have pockets of the county where the wifi is rubbish and actually there are still large groups of people that don’t have any access to any digital technology, and we have to accept that.

Provider A, Director of Nursing

Second, there was a view that remote services, which were the only option in certain periods of the pandemic, were less appropriate, if not ineffective, for some mental health conditions, such as autism and emotionally unstable personality disorders. This brings to the fore the role of technology experts (designers and programmers) and the need to ensure that devices are developed to offer optimum utility to every service user. Third, and closely related to the second point, is the ethical aspect of patient choice, which was not always guaranteed during the peak of COVID-19. The informants agreed that remote consultation was not a true substitute for in-person interaction. It was suggested, like for patients, that mental health staff, who could only attend web-based meetings during the peak of the pandemic, dearly missed the face-to-face (social) interaction with colleagues in the office:

There’s been real pros to the new approach and real limitations...I think for example, some of the personality disorder services, people with EUPD; those would have always been face to face previously, they’re now online but they’re going back to face to face and some of that works for that cohort of patients, some of it doesn’t. If you’re paranoid and schizophrenic and worried about computers, you know, your experience is going to be very different isn’t it?

Provider C, CEO

Pretty much, autism assessments as well which was something that we struggled with to start with...It’s difficult to do those assessments; it’s not ideal to do it virtually because you’re not picking up on everybody’s body language.

Provider A, CEO

Fourth, informants also highlighted the challenges with privacy, particularly for patients requiring a private “safe space” to discuss personal and confidential issues without the potential for other members of the household to overhear. For example, victims of domestic violence, children experiencing neglect, and people who are not confident in talking about their mental health issues.

Digital illiteracy was not only an issue for older service users. It was reported that some services, particularly in providers A and B, were adopting digital consultations during the pandemic for the first time, and many of their staff members needed “skilling-up” to be able to use the technology. As noted above, case study providers made concerted attempts to mitigate some of the sociotechnical challenges, including drawing on government and private sector funding support to upgrade their technology infrastructure and collaborating with voluntary organizations to address the problem of digital inequality. Finally, providers were quick in providing their staff with the requisite support—equipping them with the tools and skills needed to make the switch to digital technology and enabling staff to be accredited to conduct remote assessments. An equally important factor was the keen sense of commitment to care for patients on the part of mental health professionals during the pandemic. There was a convergence of views that mental health services have shown remarkable resilience and that staff were doing their utmost, often to the point of burnout or exhaustion, to provide optimum quality service during the pandemic.

## Discussion

### Principal Findings

We examined the perspectives of national stakeholders and staff from 4 mental health providers and found evidence that the pandemic served as a catalyst for the rapid uptake and deployment of IT in mental health care delivery during the pandemic. This finding is consistent with earlier studies on the ability of mental health providers and services to adapt and respond to the needs of service users during the pandemic, mainly through remote service provision and the use of technology [1,2,4,5,9-12,15-19]. We have also provided additional evidence on the mixed effects on the quality and efficiency of the radical switch to remote (mental health) service provision during the pandemic. Very few studies in this area had focused on the mental health sector. The study has further, perhaps for the first time, explored, using qualitative data and an STS lens, the mix of sociotechnical and system-wide factors affecting remote mental health provision in England.

### Provider’s Adaptation of Service Provision and Technology Capabilities

It has been suggested that a health care organization, being a sociotechnical system, needs to be resilient [34] not least during a public health emergency. We found that the case study providers demonstrated a remarkable resilience in responding to the needs of service users during the pandemic by rapidly adapting service provision. The mental health sector in the United Kingdom and elsewhere can potentially build on the momentum in the rollout of IT created by the pandemic [4,11,13]. When COVID-19–imposed restrictions proscribed face-to-face consultations, mental health providers were compelled to adapt their services to continue to respond to the needs of patients. Telephone and video consultations quickly emerged as the default strategy. There is evidence of improvement in the use of digital technology in mental health care delivery, following the outbreak of COVID-19, with some services adopting the use of digital platforms for the first time. Despite the progress made, the case studies have provided additional evidence that telephone communication has remained the main form of contact used by mental health providers in England, including the provision of 24/7 crisis support lines by mental health services during the pandemic [4,10,13]. Nevertheless, its effectiveness is disputed, with reports that the telephone lines have not been operational 24/7 [19]. This means that despite the reported progress, the digitalization of mental health care has remained less than optimal and that mental health services in England are still not yet at the level of digital capability that the providers would like them to be.

The case study findings revealed variability within and across provider organizations with respect to the pace at which IT was rolled out to deliver remote services. Some services that had earlier adopted remote service provision before the pandemic, notably talking therapies (Increasing Access to Psychological Therapy services), were quicker in adapting or expanding the use of digital technology. Although all providers appeared to roll out IT during the pandemic relatively quickly, we found evidence of disparities between providers in technological preparedness, with the high-performing providers, C and D, being better prepared (being a GDE and a GDE fast follower, respectively) than their low-performing counterparts, A and B. In contrast, the low-performing providers were neither a GDE nor a fast follower, and both required greater investments and more time and effort, including equipping clinicians with digital devices and the requisite training or skills to use the tools and adapt to the innovative ways of delivering services. Therefore, how soon and how much both staff and service users realized the benefits of IT during the outbreak depended largely on how advanced the provider’s technology infrastructure was (or their digital maturity).

### Impact of IT Use on the Quality and Efficiency of Mental Health Care During COVID-19

The case study findings were mixed regarding the impact of telephone and digital consultations on the quality and efficiency of service delivery during the pandemic. Conclusive evidence regarding the true impact on the quality of care is lacking [7]. Given that providers found it difficult to process routine quality measures during the peak of the pandemic, informants’ perspectives were either based on anecdotal evidence or inferred from what they saw as the benefits and drawbacks of remote service provision. However, from the case studies, the rapid rollout of IT to deliver mental health care during the pandemic appeared to have been accompanied by a number of benefits and disadvantages for patients and staff, which is consistent with the findings of other studies [1,6,11-13,16-20]. The greatest benefit was that mental health services managed to respond remotely to the needs of patients when face-to-face consultation was not possible, serving as a convenient and cost-saving strategy for interaction between patients and mental health professionals. Service efficiency, in particular, has been positively affected by the ability of mental health professionals to attend to as many patients as possible within a short period. In addition to financial savings (from reduced travel and use of office space and facilities) with some providers rethinking how to use estate facilities more efficiently, the mental health sector’s contribution to the wider environment in terms of reduced carbon emissions from less driving during COVID-19 has been unprecedented. However, linked to the major downsides of video and telephone consultations were serious ethical and clinical concerns. The concerns centered on the notion that telephone or digital technology (more so telephone) cannot be a true substitute for face-to-face consultation [10], not only because it rules out the sociopsychological utility of physical interaction but also because some study participants suggested that remote service provision could be inappropriate for some patients with certain mental health conditions such as personality disorders and autism. Concerns have also been raised about the potential for worsening inequalities stemming from digital exclusion. This brings into focus the critical underlying sociotechnical factors at play, affecting the uptake and effectiveness of IT in the delivery of mental health services and the implications for quality and patient experience.

### Determinants of IT Use in Mental Health Care Delivery—STS Framework

Mental health providers, as all health care organizations, are composed of complex macro and micro systems or subsystems, including departments, services, and units manned by humans and communities of service users—all with their dynamic and unique characteristics—and technical and other social elements [22-25,35]. Drawing on STS theory, we found that a range of factors are important for understanding the use (and optimization) of IT to deliver mental health services during and beyond the pandemic.

From the case study findings, it is evident that system-wide factors are key to IT uptake during the pandemic. The July 30, 2020, statement by the Secretary of Health and Social Care, in which he stressed the urgent need for technology in remote service provision [11], provided the national impetus and system-wide platform for a switch to digitalization of health services, including mental health. Nevertheless, the degree of prepandemic digital maturity influenced the speed with which a provider managed to switch to innovative digital options in delivering mental health services [7-13], with providers C and D being ahead of A and B in that regard. Digital maturity was, in turn, a function of historical investments in technology, which saw provider D becoming a GDE and provider C a GDE fast follower 3 years before the COVID-19 outbreak. A number of macrolevel and mesolevel factors were fundamental in enabling mental health providers, including those who were hitherto less digitally enabled, to meaningfully invest in their technology infrastructure and adopt more innovative ways of delivering services. These include new funding from the government, a more flexible approach to contracting and disbursements, donations from the private sector, and collaboration with voluntary and charitable organizations to empower service users to access digital service offers. There were concerns, however, about how providers could ensure that nationally mandated “digital governance” policy and standards are in place and being followed to protect the rights of all individuals using digital technology to access service. Digital governance at the system and provider levels, particularly regarding privacy and data protection for patients, is a fundamental aspect of the digitalization of health care service delivery [11-13].

It has been suggested that collaboration between the relevant agencies and actors in the health care system—government, Department of Health, NHS England and Improvement, NHS Digital, producers of technologies for mental health, mental health providers, and patient groups—is critical to enhancing information and digital technology use in improving mental health care delivery [22-25,35]. This could help, for example, to enhance the effectiveness of digital options through a careful design of digital tools to meet the special needs of patients with very challenging mental health conditions, such as autism and personality disorders.

Alongside the system-wide factors, a range of social and technical factors have been at play, influencing the use of information and digital technology. These factors are important in any attempt to optimize the benefits and minimize the disadvantages associated with the use of IT in mental health care. The social status of service users, notably digital poverty or exclusion, as highlighted above, has emerged as an important underlying factor of digital inequality. Providers have been mindful of this and have been making efforts to address the problem and promote digital inclusiveness within available resources and support from the system (notably provision of digital devices to service users who could not afford them and free internet cafes at strategic locations). However, this raises issues around how providers could expand and sustain such effort as a “digital library” scheme as well as have safeguarding and data protection under control [9,12,36-38].

We found that patients with mental health issues, like other members of the population, responded to the blanket national policy pronouncements around COVID-19 safety restrictions including the “stay at home order,” with many presenting when their conditions were already deteriorating. Perhaps a realization that remote service provision, which became the default option, is never a true substitute for face-to-face consultations and that some mental health cases are less suited to remote consultation might have called for a more nuanced messaging and approach to mental health patients during the peak of the pandemic. An important finding is that remote service provision or interaction means that service users and staff missed the positive element of “socializing” via face-to-face meetings, with negative implications for their well-being. It is clear that staff have become exhausted and bored with the monotony of web-based meetings. In scaling up telephone and digital consultations there is a need to pay close attention to the needs and preferences of staff and patients and services before deciding which option is best for them.

As Greenhalgh et al [9] have reported, there is an implicit dilemma or contradiction in the notion that telephone and digital solutions were available to all service users as an option. This is because not all patients were in a position to take up the offer, for example, because of technology and Wi-Fi accessibility issues or being uncomfortable with technology as a medium for receiving care. Thus, patient choice remains paramount to clinical decision-making, and it is vitally important that providers quickly adjust their mode of service provision by switching to face-to-face consultation whenever possible.

### Strengths and Limitations

The study participants included key national stakeholders; senior managers; and executive team members of case study providers, including clinicians, allowing a rich mix of perspectives to reflect both the national landscape of mental health care provision and localized service provider contexts. Another strength of this study is its exploration of the key underlying factors influencing the uptake of IT through the application of STS theory. However, this study has some limitations. First, typical of a case study of this nature, it is difficult to generalize the findings to the rest of the mental health sector in England or the United Kingdom, let alone to other health care systems. However, the involvement of national stakeholders has helped to provide some useful generic insights into the role of technology in mental health delivery during the pandemic. Second, the study was purely qualitative, drawing on the subjective views and perspectives of the study participants, without complementary quantitative data to explore causal linkages, such as between the use of technology and clinical outcomes. Third, the study was limited to senior officials at the case study sites, representatives of commissioning groups, and a very small number of patient representatives, without room for lower-level health professionals or frontline staff.

### Conclusions

The study highlights that digitally enabled remote mental health service provision has accelerated during the COVID-19 pandemic in England, with many services still heavily reliant on telephone for remote service delivery. Mixed messages emerged regarding the effects of IT on service quality and efficiency during the pandemic. Among the positive aspects is the perception that patients have generally benefited from the convenience of remote consultation when face-to-face meeting was impossible. Mental health professionals have been able to attend to many patients more rapidly than would have been possible with face-to-face consultation, saving travel time costs for both providers and service users. The major downsides of remote consultation include reduced levels of quality for people with specific types of mental health conditions requiring face-to-face contact, patient choice being compromised, and the likelihood that inequalities have been exacerbated because of a growing “digital divide.” A full assessment of the impact of IT use on the quality and efficiency of mental health care provision after the pandemic will require further and a more sustained longitudinal research. Given that telephone remains the most common means of remote consultation, there is a need for more targeted effort to support service users and health professionals (in need of such support) in catching up with the pace of digital transformation. In any future public health emergency, it is recommended that decision makers and service providers carefully consider the most appropriate service delivery option for specific services, ensuring that service provision options remain responsive to patient needs and support patient choice and retaining face-to-face provision when preferred.

## Appendix

Multimedia Appendix 1 Breakdown of study participants by organization (N=53).

Multimedia Appendix 2 Quality and efficiency of mental health care provision in a time of COVID-19: topic guides.

Multimedia Appendix 3 EMHeP COVID-19 case study codebook.

## Abbreviations

CQC: Care Quality Commission
GDE: global digital exemplar
MHCN: Mental Health Commissioners Network
NHS: National Health System
STS: sociotechnical systems

## Data Availability

To ensure anonymity and confidentiality the team had undertaken (in the Integrated Research Application System form) that data, in the form of transcribed manuscripts, will be stored securely on the university system and accessible only to members of the research team via a secure network for a period of 12 months to 3 years, following the end of data collection.
