# Supplementary material for: IT and the Quality and Efficiency of Mental Health Care in a Time of COVID-19: Case Study of Mental Health Providers in England
Source: JMIR Form Res. 2022 Dec 29;6(12):e37533. doi: 10.2196/37533 (PMC9822565; doi:10.2196/37533)
Supplement: Multimedia Appendix 1 [file formative_v6i12e37533_app1.docx]

Multimedia Appendix 1

**Breakdown of study participants by organization (N=53)**

|  | **Provider A (n=14)** | **Provider B (n=12)** | **Provider C (n=13)** | **Provider D (n=14)** |
| --- | --- | --- | --- | --- |
| Executive (board) level | CEO | CEO | CEO | CEO |
|  | Medical Director | Medical Director | Director of Finance | Medical Director |
|  | Director of Finance | Director of Finance | Director of Nursing | Chief Finance Officer |
|  | Director of Nursing |  | Director of Strategy | Director of Nursing |
|  | Director of Strategy |  |  | Chief Operations Officer |
|  | Director of Operations |  |  |  |
| Non-exec senior management | Associate Director of People | Director of Mental Health and Learning Disability_1 | Clinical Manager CAMHS^[[1]](#footnote-1)^ | Clinical Director MH Care |
|  | Associate Director of Informatics | Director of Mental Health and Learning Disability_2 | Clinical Manager Home Treatment Teams | Regional Director West |
|  | Associate Director of Operations Adults | Head of MH Inpatients | Head of Older Adults and Community MH Team | Regional Director East |
|  | Manager Older Adults | Head of Mental Health and Learning Disability | Team Manager/ Specialist Mental Health Practitioner | Clinical Director West |
|  | Manager IAPT^[[2]](#footnote-2)^ | Service Manager Home Treatment Team | Manager Older People Community MH Team | Clinical Director Adults East |
|  |  | Manager Team for Early Psychosis | Deputy Head CAMHS | Consultant Child and Adolescent Psychiatrist |
|  |  |  | Consultant Psychiatrist_1 |  |
|  |  |  | Consultant Psychiatrist_2 |  |
| Clinical Commissioning Group/Patient Representatives | CCG Informant_1 | CCG Informant_1 | CCG Informant | CCG Informant |
|  | CCG Informant_2 | CCG Informant_2 |  | Patient Rep_1 |
|  | Patient Rep | Patient Rep |  | Patient Rep_2 |

1. Child and Adolescent Mental Health Services [↑](#footnote-ref-1)
2. Improving Access to Psychological Therapies (also known as Talking Therapies) [↑](#footnote-ref-2)
