# Supplementary material for: IT and the Quality and Efficiency of Mental Health Care in a Time of COVID-19: Case Study of Mental Health Providers in England
Source: JMIR Form Res. 2022 Dec 29;6(12):e37533. doi: 10.2196/37533 (PMC9822565; doi:10.2196/37533)
Supplement: Multimedia Appendix 2 [file formative_v6i12e37533_app2.docx]

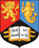


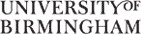


**Quality and Efficiency of Mental Healthcare Provision in a time of**

**COVID-19: Topic Guides**

**EMHeP Covid: Scoping Interview Schedule (Zoom and MS Teams)**

**1. Introductions, preamble and consent**

**2. Please briefly describe your current role**

**3. Do you have any views about changing demands for mental health (MH) services in England as a result of the Covid-19 pandemic?**

*Probe: Have you noticed whether changes in demand for MH care services have varied along ethnic lines? Please elaborate.*

**5. What in your view are the key factors required for MH trusts to cope with the added pressure because of the pandemic?**

**6. What are the ways in which MH trusts might be changing service provision as a result of Covid?**

**7. Do you know of any particular mental health service areas that are most affected in terms of increased demand because of** *Probe [if necessary]*: For example, example inpatient services, community services, early intervention for psychosis, CAMHS?

**8. Do you think the pandemic has directly affected the quality of mental health care being provided?**

- *Probe: If ‘Yes’ in which service areas do you think quality might have improved?*
- *In which service areas do you think quality might have deteriorated?*
- *Can you suggest any reasons for improvement/drop in quality?*

**9 In what ways do you think the pandemic might affect the efficiency of care in MH trusts?**

**10. What workforce issues, if any, have you seen emerging in MH trusts in the wake of the Covid-19 pandemic?**

**11. Do you think that the mental wellbeing of staff, especially frontline staff, has been affected by the pandemic? How?**

- What implications do you think this would have for the quality of service provision?

**12. How well do you think the existing technology infrastructure has enabled different MH trusts to cope with the demands during the peak of the pandemic?**

- *How well do you think different MH trusts have been using digital technology to maintain or improve the efficiency and quality of the service they provide for patients?*
- *What are some of the challenges that MH trusts might be faced with in the use of technology to provide quality and efficient service that is Covid-secure?*
- *Can you suggest any particular challenges that some patients might be faced with around the use of digital technology during the pandemic?*

**13. What else do you think MH trusts are doing to accelerate new ways of working and innovation?**

**14. Do you think the relationships between MH trusts and commissioners, in terms of contracting arrangements, have changed during the pandemic? And to what effect?**

**15 Do you think relationships with other partners have changed?**

**16. What are the prospects for effective joint decision-making and joint use of resources around integrated service delivery in light of the shift to Integrated Care Systems?**

**17. From your perspective what could have been done differently at national level in terms of pandemic disaster preparedness in relation to MH services.**

**18. From your perspective what lessons could be learned for future public health emergencies with specific focus on mental healthcare provision?**

**19. Is there anything else you would like to add?**

**Conclude interview**

- Any questions?
- Thanks

**COVID-19: Case Study Topic Guide**

**EMHeP Covid: Interview Schedule (Zoom and MS Teams)**

**1. Introductions, preamble and consent**

I’m Fred Konteh, Research Fellow at the UoB. We’re exploring the impact of Covid-19 on Quality and Efficiency of mental health provision.

**2. Please describe your current role and responsibilities**

**3. Please describe how much has changed in terms of demands of mental health services relative to the service provision capacity at the Trust following the outbreak of Covid.**

- To what extent has the Trust had to change or adapt its strategy of service provision as a result of Covid?
- Have any services been redesigned for patients and users? How?

**4. What specific service areas have been most affected in terms of demands and the quality and efficiency of service provision? (For example inpatient services, community services, early intervention for psychosis, CAMHS)?**

- Has the Trust changed strategy for specific services in the wake of Covid-19 (such as the ones mentioned above)?

**5. Which specific service areas have been least affected?**

**6. In what ways has COVD19 directly affected the quality of care that you provide? Are there any specific patient outcome/indicators, you are aware of, that have been seriously affected and to what extent?**

**7. Has the ethnic composition of people seeking care from your service changed or differed in the wake of the pandemic?**

**8. In what ways has the COVD19 pandemic directly affected the efficiency of care that you provide?**

**9. Have there been any emerging workforce issues as a result of Covid-19 pandemic?**

- [If any] How have you dealt with such challenges as a MH service provider?

**10. How much added work pressure, if any, do you think the pandemic has brought on staff in your trust? How well have your staff been coping with the pressure?**

**11. Has the mental or psychological wellbeing of some of your staff been affected by the pandemic? How?**

- How, if at all, did this affect the quality of service provision?
- How has the trust been dealing with the situation?

**12. To what extent was ethnicity a factor in how the pandemic has affected staff members – their psychological or mental well-being and how they have generally responded to the shocks of Covid-19?**

**13. How much did the Trust’s existing technology infrastructure cope with the demands for MH care provision especially during the peak of pandemic?**

- How much did the use of digital technology come into play in your effort to maintain efficiency and quality service provision for patients?
- How adequate was the technology infrastructure and facilities for staff and service users alike? What challenges has the Trust had to deal with in this respect? How did you handle the challenges (if any)?
- Could the Trust have done anything differently in terms of optimising the use of technology during the crisis?

**14. What else did the Trust do in terms of seizing opportunities to accelerate new ways of working and innovation?**

**15. Have you experienced the diversion of mental health resources to support Covid-19 patients, including the use of mental health beds and staff being seconded?**

- Please elaborate

**16. Please explain whether the Trust has had to adapt payment and contracting approaches during the pandemic e.g. re-introduction of block contracts, and whether the perceived role of commissioning for quality and efficiency changed.**

**17. Please explain whether relationships and partnerships with the Trust’s partners have had to change in any way during the pandemic and to what effect?**

**18. To what extent has the pandemic brought about changes, if any, in collaboration and coordination between your Trust and others around integrated service delivery?**

**19. What’s your view about the setting up of joint committees within the framework of an Integrated Care System?**

**20. What are the prospects for effective joint decision-making and use of resources around integrated service delivery in the near future?**

**21. From your perspective what (if anything) could have been done differently by your Trust in terms of pandemic disaster preparedness?**

**22. From your perspective what lessons could be learned particularly for future health public health emergencies with specific focus on mental healthcare provision?**

**23. Is there anything else you would like to add?**

**Conclude interview**

- Any questions?
- Thanks
