# Supplementary material for: IT and the Quality and Efficiency of Mental Health Care in a Time of COVID-19: Case Study of Mental Health Providers in England
Source: JMIR Form Res. 2022 Dec 29;6(12):e37533. doi: 10.2196/37533 (PMC9822565; doi:10.2196/37533)
Supplement: Multimedia Appendix 3 [file formative_v6i12e37533_app3.docx]

Multimedia Appendix 3

EMHeP COVID-19 Case study Codebook

Codes

| Name | Description |
| --- | --- |
| 1. Community transformation funding | Where the Provider is said to benefit from community transformation funding |
| 1. Contracting arrangements | Whether the arrangements around contracting (allocation of funds) between clinical commissioning groups and providers have changed during COVID, and how |
| 1. Coping with demand and pressure on services | How trusts and services coped with the pressure to deliver service during COVID-19 |
| 3.1 24 hour helpline activated |  |
| 3.2 Being positioned/prepared to be able to cope |  |
| 3.3 Bespoke training provided |  |
| 3.4 Closing some wards with no activity |  |
| 3.5 Closing wards due to staffing issues |  |
| 3.6 Community services struggling to cope |  |
| - 1. Demand and capacity modelling by Trust | Where demand and capacity modelling is mentioned as strategy adopted during COVID-19 |
| 3.8 Discharging too quickly | Early discharge of patients |
| 3.9 Focus on safety |  |
| 3.10 Hybrid working |  |
| 3.11 Increased investments |  |
| 3.12 Low sickness rate |  |
| 3.13 Offering staff financial incentive |  |
| 3.14 Out of area placement |  |
| 3.14.1 No out of area concerns |  |
| 3.15 Remote vs face to face service |  |
| 3.16 Removal of beds because of COVID-precautions |  |
| 3.17 Routine CPD suspended | Continuous professional development |
| 3.18 Staff showing resilience |  |
| 3.19 Stepping down services |  |
| 3.20 Stepping up new services |  |
| 3.21 Telephone consultation |  |
| 3.22 Trust not hard-hit in the first wave |  |
| 3.23 Wards being closed because of COVID breakout |  |
| 4. Demand for services | Demand for MH services during COVID |
| 4.1 Acuity or severity of cases |  |
| 4.2 Bed capacity |  |
| 4.3 Delayed effect |  |
| 4.4 Drop in referrals |  |
| 4.5 Fluctuating demand |  |
| 4.6 Increased demand |  |
| 4.7 New presentations |  |
| 4.8 Non-reporting due to perceived stigma |  |
| 4.9 Patients being responsive to service adjustments |  |
| 4.10 Patients staying away because of COVID |  |
| 4.11 People being confused and anxious |  |
| 4.12 Pressure on services |  |
| 4.13 Service areas least affected |  |
| 4.15 Service areas most affected |  |
| 4.15.1 Autism |  |
| 4.15.2 CAMHS |  |
| 4.15.3 Community services again |  |
| 4.15.4 Difficulty in delineating services affected |  |
| 4.15.5 Eating disorders for children and young people |  |
| 4.15.6 Eating disorders for older patients |  |
| 4.15.7 EUPD |  |
| 4.16 Inpatient services |  |
| 4.17 Learning disability |  |
| 4.18 Sicker patients, severity of cases |  |
| 4.19 Variable demand based on services |  |
| 5. Effects on quality | How COVID-19 affected the quality of service |
| 5.1 Adverse effect on quality |  |
| 5.2 Coincidental increase in demand |  |
| 5.3 Difficult to measure impact on quality |  |
| 5.4 Impossible to follow social prescribing |  |
| 5.5 Inequalities exacerbated |  |
| 5.6 Less focus on quality |  |
| 5.7 Measuring effect on quality |  |
| 5.8 Patient experience |  |
| 5.9 Patients providing positive feedback |  |
| 5.10 Quality affected through COVID infection |  |
| 5.11 Quality affected through staff burnout |  |
| 5.12 Quality not affected |  |
| 5.13 Rushing patient through the door |  |
| 5.14 Services adversely affected |  |
| 5.15 Variable effect on quality |  |
| 6. Efficiency and productivity | How the efficiency of service delivery was affected (including finance and service efficiency) |
| 6.1 Finance efficiency difficult to  Determine |  |
| 6.2 Financial gains |  |
| 6.3 Impact on Estate |  |
| 6.4 Impact on the Environment |  |
| 6.5 No financial gains |  |
| 7. Ethnicity as a factor | To what extent did ethnicity become a factor both among staff and service users? |
| 7.1 Asian ethnic minority staff severely affected |  |
| 7.2 Ethnicity not a factor for staff |  |
| 7.3 Ethnicity not a factor for patients |  |
| 8. Extraneous or external factors | Any factors discussed to do with the external environment (the general health system) |
| 9. Geography of study site | Where geography is mentioned, including the remoteness of some communities |
| 10. Impact on leadership | How leadership style has been affected |
| 11. Impact on technology | How the use of technology has been affected |
| 11.1 Digital inequality |  |
| 11.2 Digital first or by default |  |
| 11.3 Embracing MS Teams |  |
| 11.4 Enablers |  |
| 11.5 Good infrastructure |  |
| 11.6 Inadequate infrastructure |  |
| 11.7 IT governance and security issues |  |
| 11.8 Limitations of digital service |  |
| 11.9 No worries about finance or funding |  |
| 11.10 Positive technology effects |  |
| 11.11 Staff being trained |  |
| 11.12 Technology constraints |  |
| 11.13 Young people being negative about technology |  |
| 12 Innovation | Where innovation is discussed |
| 12.1 Service redesign or adjustments |  |
| 12.2 Speed of change |  |
| 13. Key lessons | The key lessons from COVID-19 and the response of mental health providers |
| 13.1 Appreciating the value of mental health |  |
| 13.2 Assessing impact on the most vulnerable population |  |
| 13.3 Being able to take tough and urgent decisions |  |
| 13.4 Better use of data for patient quality |  |
| 13.5 Blended or hybrid approach |  |
| 13.6 Caring for or valuing staff |  |
| 13.7 Changing entrenched beliefs and assumptions |  |
| 13.8 Collaboration is key |  |
| 13.9 Demand and capacity modelling as a system |  |
| 13.10 Flexible working |  |
| 13.11 Focus on staff wellbeing |  |
| 13.12 Good leadership is highly important |  |
| 13.13 Greater consciousness about mental health |  |
| 13.14 Greater staff engagement at service level |  |
| 13.15 Guidelines not sufficient |  |
| 13.16 Infection control |  |
| 13.17 Less bureaucratic |  |
| 13.18 Listening to top scientists and clinicians |  |
| 13.19 Not being well prepared |  |
| 13.20 Rapid transformation |  |
| 13.21 Services focusing on core business |  |
| 13,22 The value of IT in crisis time |  |
| 13.23 Trusting staff to be professional in flexible working |  |
| 13.24 Unclear policy messages about the MH sector |  |
| 13.25 What could have been done differently |  |
| 14. Relationships | How relationships were affected, including internal and external relationships |
| 14.1 Difficulty with internal relationships |  |
| 14.2 Good internal relationships |  |
| 14.3 Good system-wide relationships |  |
| 14.4 Poor or difficult system-wide relationships |  |
| 14.5 Virtual Teams meetings |  |
| 15. Staff redeployment | Instances in which staff were being redeployed |
| 16. Staff Wellbeing | How staff wellbeing was affected and addressed by providers |
| 16.1 Emotional pain from loss of colleagues or close ones |  |
| 16.2 Impact on morale and resilience |  |
| 16.3 Long term effects |  |
| 16.4 Occupational health |  |
| 16.5 Service or team level support |  |
| 16.6 Staff being risk-assessed |  |
| 16.7 Staff missing physical & social interaction |  |
| 16.8 Staff not accessing offer |  |
| 16.9 Staff were unequally affected |  |
| 16.20 Trust level support |  |
| 17. Supply factor | Making services available to service users |
| 17.1 Services being closed |  |
| 18 System-wide collaboration | Level of collaboration and cooperation between MH providers and partners (including acute providers, CCG and the private sector) |
| 19. Use of data in decision making | How data was used to influence decision-making |
| 20. Views on Integrated Care Service | What informants think about the impact of COVID-19 on the evolution of ICS |
| 20.1 Fear about organisations being too 'protective' |  |
| 20.2 Less optimistic about ICS |  |
| 20.3 Optimistic about ICS |  |
| 21. Waiting lists | Impact of COVID-19 on waiting lists |
| 22. Workforce issues | Other issues relating to the workforce |
| 22.1 Issues around PPE |  |
| 22.2 Mitigating staff shortage |  |
| 22.3 Sickness related staff shortage |  |
| 22.4 Staff being hit by COVID |  |
| 22.5 Staff overworked and exhausted |  |
| 22.6 Staff shortage |  |
| 22.7 Staff struggling with new mode of work |  |
